# History of a Case of the Infantile Disease Generally Called the Purulent Ophthalmia, with Observations

**Published:** 1808-07

**Authors:** Thomas Morrison

**Affiliations:** Surgeon, Dublin


*History of a Case of the Infantile Disease generally called the* PURULENT OPHTHALMIA, *with Observations; by* THOMAS MORRISON, *Surgeon, Dublin.*

MUCH time, attention and study are required to advance any branch of human knowledge to perfection; and it seems to be the endeavour of every age, not only to profit by the improvements of their ancestors, but to discover and rectify their errors.

In new-born infants a peculiar inflammation of one or both eyelids is sometimes met with by Practitioners in Midwifery, which has of late years received the appellation of the *purulent ophthalmia;* distinguished from every other species affecting that membrane, by the enormous quantity of yellow matter secreted from the eye-lids, and the extreme swelling thereof. It often appears on the day after birth, but more frequently on the third or fourth day succeeding to that event; and is supposed, by the majority of authors, to arise from the infant being imprudently exposed to a cold atmosphere; yet, many will justly question the above causes, and *others lately assigned,* for this formidable complaint; but careful observers will join with me in opinion, that it is accompanied with such appearances, as to make it manifest to them, that it derives its origin from a different source.

 It

It frequently happens in this deplorable complaint, that medical and surgical aid is not solicited, until it has nearly run its period, and done every possible mischief: for mid-wifes, too ignorant to distinguish its real cause, are apt to procrastinate, as confounding it with those simple purulent cases that subside spontaneously in a few days.

The ingenious Mr. Ware, and others, who have written professedly upon the diseases of the eye, entertain no suspicion of this complaint having resulted from a venereal taint. Dr. Edmonston, in his treatise on the ophthalmia, is of the opinion, that it is connected with a loaded and oppressed state of the stomach and intestines; I am ready to allow such an occurrence does often accompany the diseased eye, and, when it occurs, may pretty generally aggravate the symptoms; but the disease in question, I am decidedly of opinion, is produced by a venereal cause: the following circumstance suggested it to me. I was consulted some years ago in the case of Mrs. N's child, who ultimately lost one eye from an attack of this disease. On my first days of attendance, Mrs. N. drew my attention to an affection which existed previous to her parturition, and had continued subsequently thereto; this complaint was unequivocally a case of the venereal kind, which had been suspended in its action by advanced gestation; the coincidence of a similar discharge in the mother and fœtus excited my particular attention, and it occurred to me, that the eyes of an infant, during their passage through a vagina where such a purulent virus was present, would be liable to receive the disease in question by contact between the matter and the eyes of the infant; this; as well as eight cases which since occurred to me in practice, assumed the following appearances.

The disease first announces itself by a redness of the eye-lids, which in thirty-six or forty-eight hours, swell to a size of so great a magnitude as to prevent their being separated without the utmost difficulty; shortly after, a profuse discharge of yellow matter succeeds, which, if the eye-lids can be separated, will be seen to cover and entirely overspread the globe of the eye.

In general, both eyes are attacked in a similar way; and, in obstinate cases, whenever the infant cries, the internal surface of the eye-lids are turned outward, which is also the condition of the parts, when a forcible attempt is made to separate them with the fingers. *This seems most generally the state of the palpebræ*; and though they should be restored by the attendant to their natural situation, yet yet they do not long retain it, but return very quickly to their former everted state.

In such cases, the tumefaction of the eye-lids occasions a constriction of their ciliary edges; by means of which, the retained matter is prevented from running off, and, its lodging between the eye-lids and the globe of the eye, serves still further to increase the inflammation; and is also one of the frequent causes of obstinate ulcers, staphiloma, and specks, which often partially, and sometimes totally, cover the pupil.

Taking into consideration that the foregoing disease puts on appearances somewhat similar to a gonorrhœa, I was induced to try a practice not very dissimilar, *by ablution and injection*; and I can confidently state, that it answered my most sanguine expectations. My advice in those cases is, to sponge the eyes six or eight times in the day with tepid milk and water, so as to remove the contained matter; and immediately after each ablution, to use a lotion composed of the following ingredients: Take of rose water, eight ounces, dissolve therein two grains of the corrosive muriate of quicksilver, (Pharmacopœia Dubliniensis) well triturated, and add of the liquor of the subacetate of litharge one drachm: it will be also adviseable to inject frequently with a syringe, some of this lotion under the eye-lids. Moreover, I generally combine with the external ablution, a few internal medicines; those which I have preferred, were merely composed of the eighth part of a grain of sub-muriated quicksilver and five of prepared egg-shells, to be administered in a little panado or gruel, each night, to complete the cure.

### OBSERVATIONS.

I may be told that this case rests on too slender grounds, to induce the reader to believe that the fact is established in a perfectly unequivocal manner; and that giving such a statement to the world, must tend to disturb domestic tranquillity. Few persons feel more for the peace of the community than I do, consequently, every means ought to be observed, and every care taken, to obviate such difficulty; but as the cause of truth ought to be impartially respected, in giving cases of this nature to the public, I will introduce a few other facts, which must convince the most wavering mind, as to its having a venereal origin, and that the virus became locally applied to the tarsæ, when the fœtus was issuing into the world.

We must beg leave to call to the recollection of the reader, the different effects produced by the application of venereal matter to an absorbing surface, and of that same matter when applied to a secreting or mucous membrane; in the former, a chancre and all its train of consequences are produced; in the latter, we find a profuse discharge uniformly takes place, which, in cases of the eye, commonly ends in total blindness; and in gonorrhœa, terminates in gleet, strictures of the urethra, or fistula in perineo.

To confirm the opinion I early entertained of the real nature of this complaint, I was led to adopt a very bold experiment: a little matter was applied, that issued from the eyes of Mrs. N's child, to an abraded surface on the back of the hand of a young surgeon; the result was, a genuine venereal ulcer was produced, and to subdue which, the most steady administration of mercury became necessary. This case was seen by a very discerning professional gentleman, who perfectly coincided with me in opinion, that it was venereal.

Finally, every one of the females who were delivered of the aforementioned children, were fully satisfied of their having received local venereal affections, two or three months before parturition, and were treated accordingly, with the usual success.

